# Non-thermal atmospheric plasma ameliorates imiquimod-induced psoriasis-like skin inflammation in mice through inhibition of immune responses and up-regulation of PD-L1 expression

**DOI:** 10.1038/s41598-017-15725-7

**Published:** 2017-11-14

**Authors:** Yun Sang Lee, Myung-Hoon Lee, Hang-Jun Kim, Ho-Ryun Won, Chul-Ho Kim

**Affiliations:** 1 Department of Otolaryngology, School of Medicine, Ajou University, Suwon, Republic of Korea; 2 Department of Molecular Science and Technology, Ajou University, Suwon, Republic of Korea

## Abstract

Plasma medicine is an emerging novel therapeutic field. It has been reported that plasma can kill bacteria, promote wound healing and induce apoptosis of tumor cells. However, the effects of plasma on immune cells and immune related skin diseases have not been well studied. In this study, we demonstrated that non-thermal atmospheric plasma (NTP) treatment could inhibit psoriasis-like skin inflammation in mice. NTP treatment in imiquimod-induced psoriasis-like mouse skin inhibited increases in epithelial cell thickness and expression of pro-inflammatory molecules compared to ones without the NTP treatment. In addition, differentiation of Th17 cells, an important cell type for pathogenesis of psoriasis, was inhibited in the NTP-treated mouse lymph nodes. It was also demonstrated that liquid type plasma (LTP), which is also known as indirect plasma, inhibited Th17 cell differentiation *in vitro*. Other *in vitro* experiments showed that LTP inhibited bone marrow-derived dendritic cell activation. Interestingly, LTP enhanced PD-L1 expression in HaCaT cells, suggesting that NTP may inhibit unwanted over-activation of T cells through increased PD-L1 expression. Taken together, these results suggest that NTP may be used in treatment of CD4+ T cell-mediated autoimmune diseases such as psoriasis.

## Introduction

Psoriasis is a chronic inflammatory skin disorder, and its histological characteristics are epidermal hyperplasia, increased angiogenesis and immune cell infiltration^1,2^. Although the pathogenesis of psoriasis is not fully understood, many evidences suggest that Th17 cell is a major player in the pathogenesis of psoriasis^3,4^. Naïve CD4^+^ T cells can be differentiated into Th17 cells *in vitro* through culture with IL-6 and TGF-β^5,6^, and IL-23 is important for the maintenance or survival of Th17 cells^7^. Thus, the expression of genes involved in the Th17 cell differentiation is increased in human psoriasis lesions^8,9^. Experiments using a mouse model of psoriasis also show that Th17 cells and related cytokines are involved in psoriasis pathogenesis^10,11^. Therefore, it has been proposed that targeting IL-17 or its related cytokines may be an effective treatment for psoriasis. Indeed, anti-IL-12/23p40 antibody downregulates psoriasis-related cytokine and chemokine gene expressions in psoriasis patients^12^. It has also been reported that human anti-IL-17A antibody can effectively treat psoriasis during clinical trials, which confirms that the IL-17/IL-23 axis is a good target for psoriasis treatment^13^. It was also recently reported that PD-L1 is involved in the pathogenesis of psoriasis. PD-L1 expression is decreased in psoriatic epidermis compared to normal epidermis, suggesting that PD-L1 expression is necessary to inhibit the pathogenesis of psoriasis^14^. In addition, recombinant PD-L1 could ameliorate psoriatic inflammation in imiquimod-induced psoriatic mouse skin^15^.

Th17 cells are involved not only in psoriasis but also in other autoimmune diseases, including experimental autoimmune encephalomyelitis, collagen-induced arthritis, inflammatory bowel disease, and uveitis^16–19^. PD-L1 is also critical to inhibiting autoimmune diseases through suppression of CD4+ T cell activation. Therefore, anti-IL-17 antibody, anti-PD-1 antibody and/or recombinant PD-L1 therapies for psoriasis treatment might be used to treat other autoimmune diseases^20^. However, these therapies could cause risk of infections or cancers because the therapies inhibit hosts’ defense immune systems. Thus, these therapies may not be used for psoriasis patients with infections and/or cancers, and this limitation forces the development of a novel therapy to treat these patients.

Plasma is referred to as the 4th state of matter, and it consists of electrons, ions and reactive species. The medical term “plasma” was coined by Irving Langmuir because “plasma” in physics is analogous to the plasma that is ionic liquids in medicine^21^. However, the use of plasma in medical fields was not popular. Recently, many researchers tried to use plasma in the fields of biology and medicine, and various evidence supports that non-thermal plasma (NTP) can regulate many biological responses and be used to treat diseases. For example, NTP treatment of cancer cells can inhibit cancer cell migration and invasion and induce apoptosis of cancer cells^22–24^. It was also reported that plasma can be used in wound healing^25–27^, tooth bleaching^28^, muscle regeneration^29^, and atopic dermatitis^30^. Due to these accumulating reports, plasma medicine is an emerging technology in biomedicine. However, the effect of NTP on autoimmune diseases such as psoriasis has not been well studied.

In this study, we investigated whether NTP treatment can inhibit imiquimod-induced psoriasis-like skin inflammation in mice. Our results showed that NTP treatment can treat psoriasis-like skin inflammation through inhibition of CD4+ T cell differentiation, suppressed pro-inflammatory responses, and induction of PD-L1 expression. These results imply that NTP can be used for the treatment of immune-related inflammatory skin diseases.

## Results

### NTP treatment inhibits imiquimod-induced psoriasis-like skin inflammation in mice

**Figure 1:**
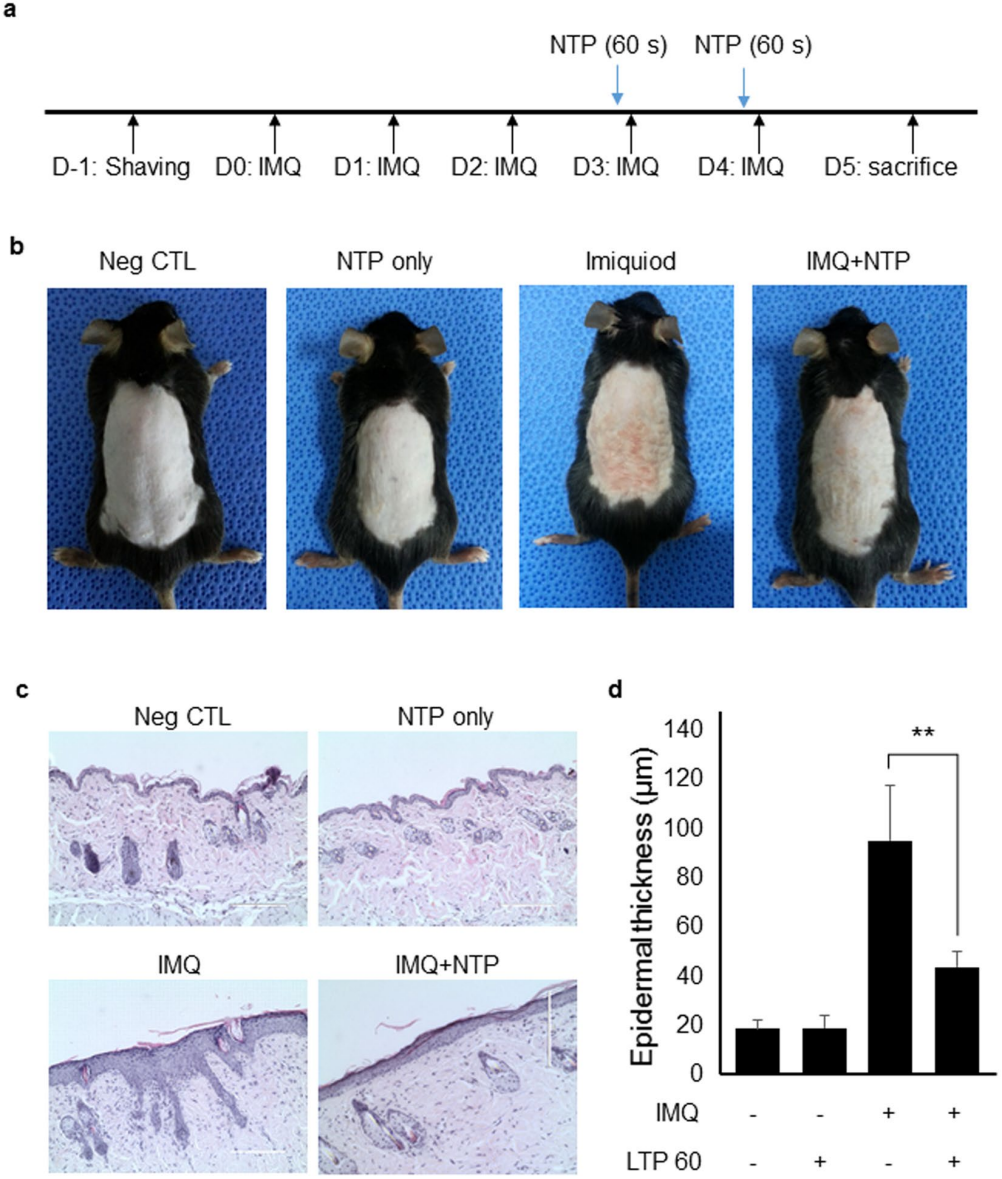
NTP treatment ameliorates imiquimod-induced psoriasis-like skin inflammation in mice. (**a**) The experimental scheme for imiquimod-induced psoriasis-like skin inflammation in mice. Imiquimod was applied onto the shaved back skin every day for 5 days. The mice were treated with NTP on days 3 and 4. (**b**) Psoriasis-like skin inflammation was induced and NTP treatment inhibited the psoriasis-like skin inflammation in mice. (**c**) H&E staining of the mouse back skin. (**d**) NTP treatment reduced epidermal thickness. The thickness was measured in several regions of the skin section. The epidermal thickness does not include hair follicles. n = 5. **P < 0.005. Bar = 100 µm.

Psoriasis-like skin inflammation was induced through application of imiquimod in the mouse back skin and NTP was treated for 60 second on day 3 and day 4 to investigate the effect of NTP on the pathogenesis of psoriasis (Fig. 1a). Figure 1b showed that NTP treatment could ameliorate imiquimod-induced psoriasis-like skin inflammation. We confirmed vehicle cream and N_2_ gas did not affect skin inflammation (Supplementary Figure 1a). Histology and gene expression of cytokines and chemokines were also not affected by vehicle cream and N_2_ gas exposure (Supplementary Figure 1b–d). In addition, H&E staining of sections from the mouse skin showed that NTP treatment decreased epidermal thickness in imiquimod-stimulated mouse skin compared to imiquimod-stimulated mouse skin without NTP treatment. However, NTP treatment in normal mouse skin did not affect skin tissues (Fig. 1c and d), suggesting that NTP may not induce side effects in normal skin tissues.

### Immune cell infiltration was suppressed by NTP treatment in imiquimod-induced psoriasis-like mouse skin inflammation

**Figure 2:**
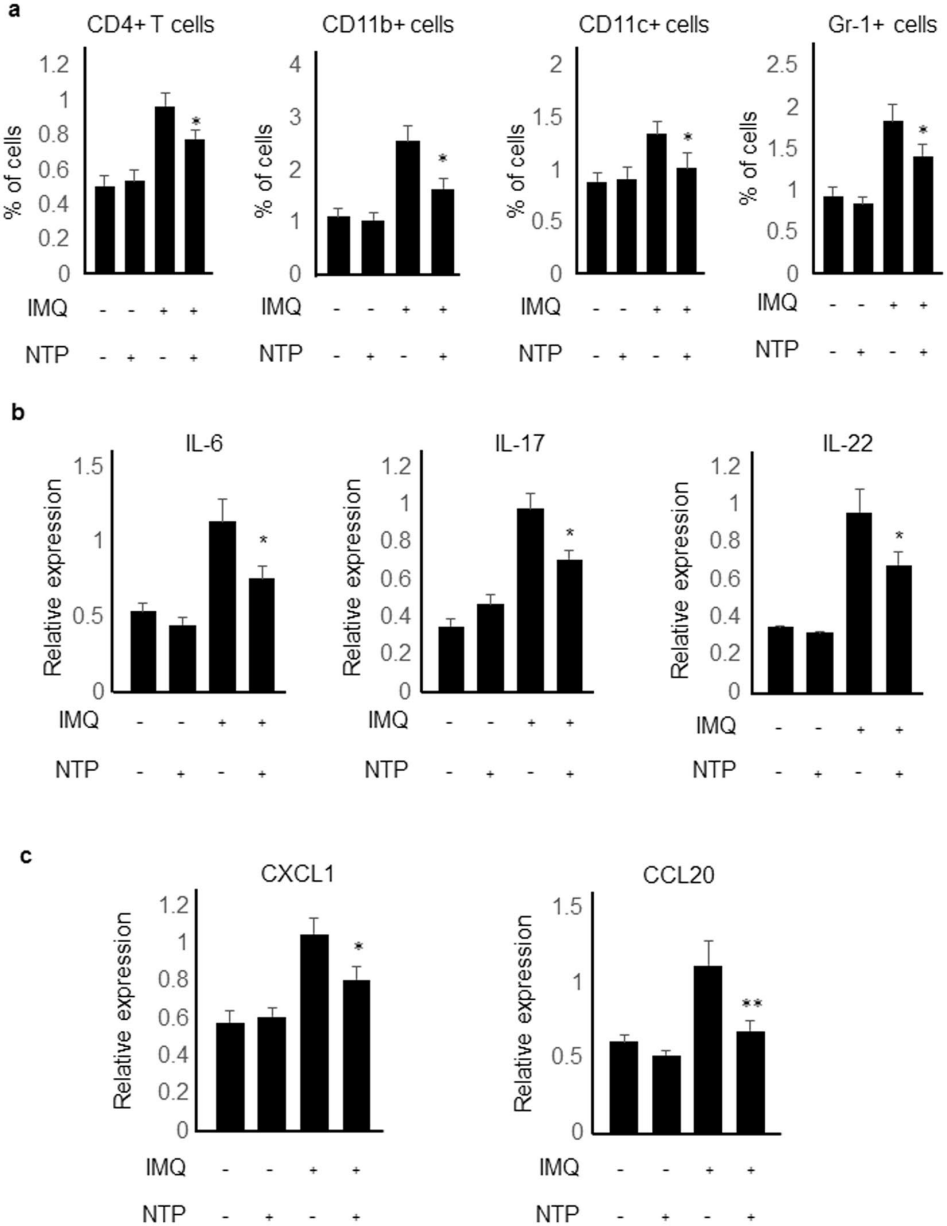
Immune cell infiltration, cytokine and chemokine expression in the mouse skin. (**a**) NTP treatment inhibited immune cell infiltration in imiquimod-induced psoriasis mouse skin. n = 5 (**b**) Psoriasis-related pro-inflammatory cytokine expression, and (**c**) psoriasis-related chemokine expression in mouse skin were determined by real-time PCR. n = 5. *P < 0.05. **P < 0.005.

To determine whether NTP could inhibit immune cell infiltration in imiquimod-induced psoriasis-like skin inflammation, single-cell suspensions from the mouse skins were analyzed by flow cytometry. CD4+ T cells, CD11c+ cells, CD11b+ cells, and Gr-1+ cells were recruited into the imiquimod-treated mouse back skins and NTP treatment suppressed the immune cell recruitment into the back skins (Fig. 2a). Cytokine and chemokine expression levels in the mouse skins were also determined because expressions of IL-6, IL-17, IL-22, CCL20 and CXCL1 were involved in the pathogenesis of psoriasis. Expression of the cytokines and chemokines were increased in the imiquimod-treated mouse skin more than double compared to the negative control and NTP-only treated mouse skins, and NTP treatment in the imiquimod-stimulated mouse skin inhibited psoriasis-related cytokine (Fig. 2b) and chemokine gene expressions (Fig. 2c). Thus, alleviation of imiquimod-induced psoriasis-like skin inflammation by NTP treatment might be through, at least partially, the inhibition of pro-inflammatory cytokine and chemokine gene expressions, resulting in inhibition of immune cell infiltration in the mouse skins.

### NTP treatment inhibits Th17 cell differentiation in the imiquimod-stimulated mouse lymph nodes

**Figure 3:**
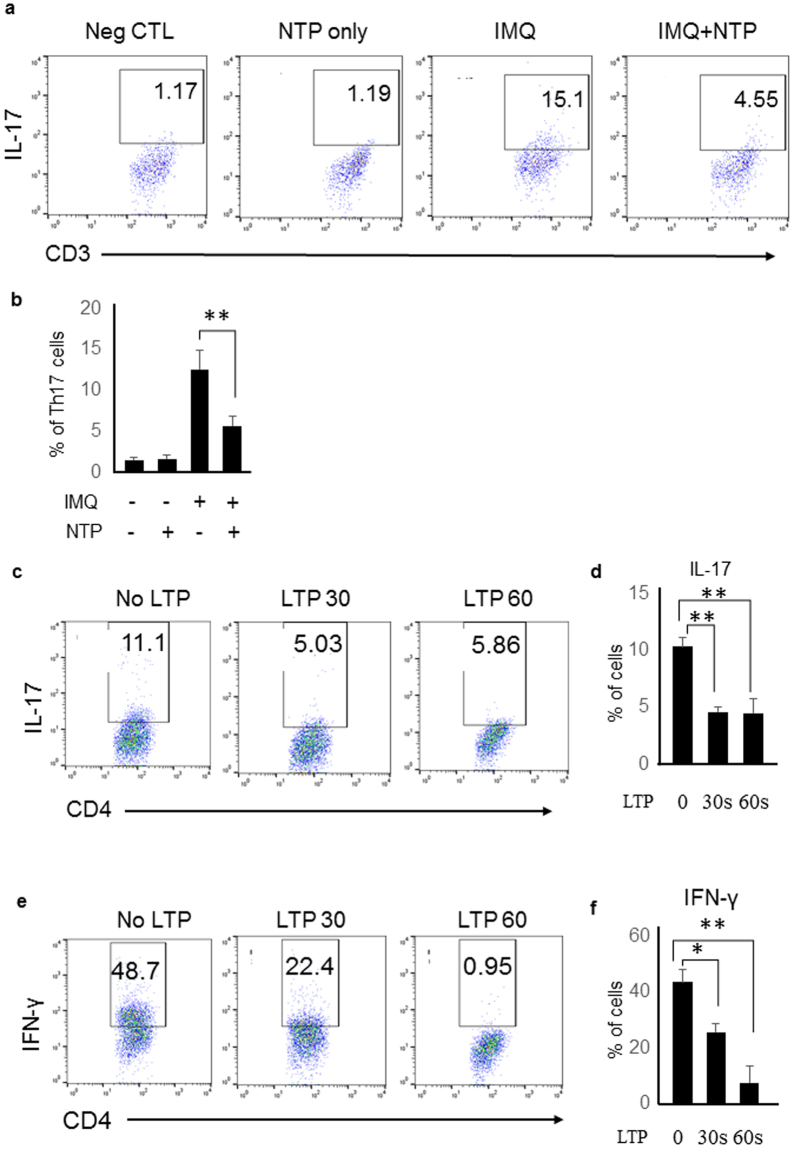
NTP treatment inhibited Th17 cell differentiation *in vivo* and *in vitro*. (**a**) Imiquimod induced Th17 cell differentiation in the mouse draining lymph node and NTP treatment inhibited Th17 cell differentiation. (**b**) Percentage of Th17 cells were represented as a graph. IMQ; Imiquimod, NTP: Non-thermal atmospheric plasma. LTP treatment inhibited (**c**) Th17 and (**e**) Th1 cell differentiation during *in vitro* differentiation. (**d**) Th17 cell percentage, and (**f**) Th1 cell percentage were represented as a graph from *in vitro* experiments. Three independent experiments were performed. *P < 0.05, **P < 0.005.

Many reports suggested that Th17 cells are important for the pathogenesis of psoriasis. Thus, we determined whether NTP treatment could suppress Th17 cell differentiation in the mouse lymph nodes of the imiquimod-stimulated mice. Application of imiquimod in the mouse back skin increased Th17 cell population in the draining lymph nodes, but the Th17 cell differentiation was inhibited in the lymph nodes of NTP-treated mice (Fig. 3a and b). To confirm the results, we performed *in vitro* experiments. Naïve CD4+ T cells differentiated into Th17 cells *in vitro* using the Th17 cell differentiation condition, in which naïve CD4+ T cells were cultured in the RPMI media containing ant-CD3 antibody (10 µg/ml), anti-CD28 antibody (5 μg/ml), IL-6 (10 ng/ml), TGF-β (5 ng/ml), anti-IFN-γ antibody, and anti-IL-4 antibody. Th17 cell differentiation was suppressed when naïve CD4+ T cells were cultured under a Th17 cell differentiation condition in the NTP-treated RPMI media (LTP) (Fig. 3c and d). Th1 cell differentiation was also suppressed when CD4+ T cells were cultured in LTP during differentiation (Fig. 3e and f). Thus, these results imply that plasma might inhibit psoriasis-like skin inflammation in mice through suppression of CD4+ cell differentiation.

### LTP affects the expressions of activation markers and cytokines in activated bone marrow-derived dendritic cells (BMDCs)

**Figure 4:**
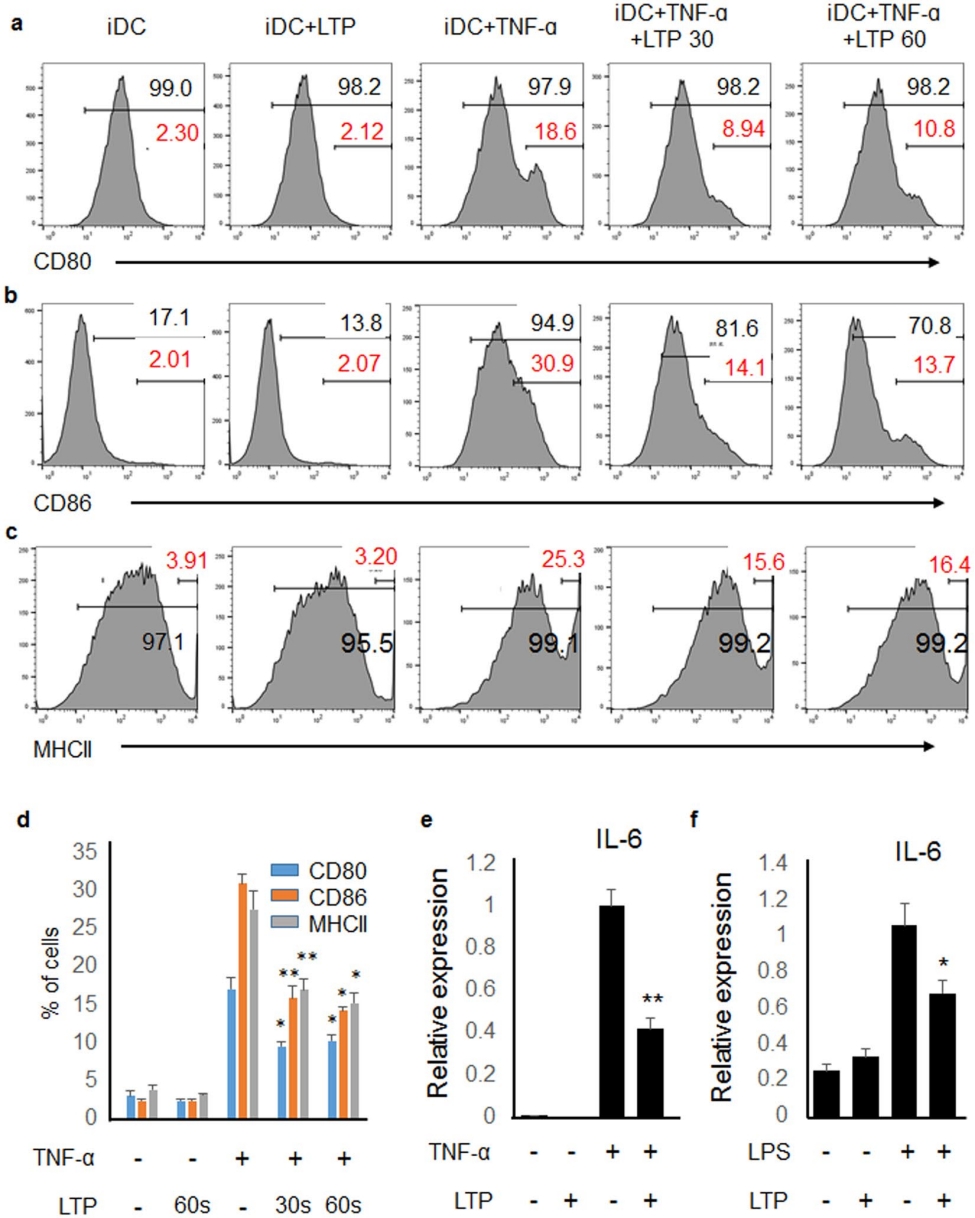
LTP treatment inhibited activation of BMDC. (**a**) CD80, (**b**) CD86, and (**c**) MHCII are activation markers of BMDC. TNF-α treatment activated BMDC and LTP suppressed the expression of activation markers. (**d**) Percentage of CD80^high^, CD86^high^, MHC II^high^ cells were represented as a graph. LTP treatment into (**e**) TNF-α-stimulated or (**f**) LPS-stimulated BMDCs inhibited IL-6 gene expression. Three independent experiments were performed. *P < 0.05, **P < 0.005.

Activation of dendritic cells (DCs) is required for the CD4+ T cell activation and differentiation. Thus, we investigated whether plasma affects DCs using bone marrow derived dendritic cells. BMDCs were generated as described in the Materials and Methods, and the BMDCs were activated with TNF-α (20 μg/ml) because TNF-α is involved in the pathogenesis of psoriasis. As shown in Fig. 4a–c, TNF-α stimulation enhanced the expression of activation markers, which are CD80, CD86, and MHCII in BDCM. However, LTP suppressed the activation marker expression. The percentage of cells which express highly activation markers were represented as a graph in Fig. 4d. We also determined the cytokine expression from BMDCs. The expression of pro-inflammatory cytokine in TNF-α-stimulated BMDC increased, but LTP treatment inhibited IL-6 expression in TNF-α-stimulated BMDC (Fig. 4e and Supplementary Figure 3). Consistently, pro-inflammatory cytokine expression was also induced in LPS-stimulated BMDC, and LTP treatment inhibited the induced expression (Fig. 4f and Supplementary Figure 4). These results suggest that plasma might negatively regulate DC activation, which results in inhibition of T cell activation and differentiation.

### LTP treatment inhibits pro-inflammatory responses in HaCaT, keratinocyte cell line

**Figure 5:**
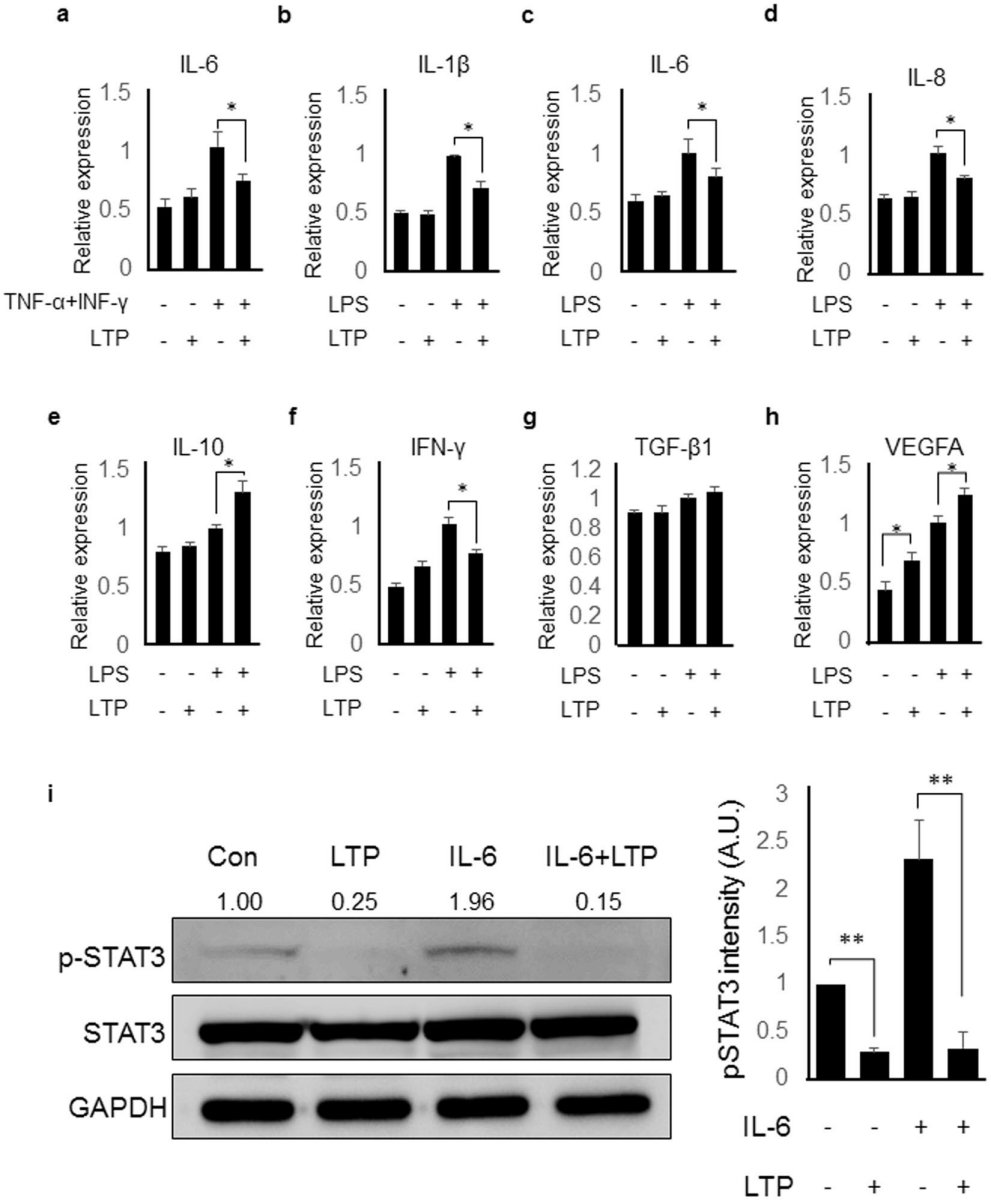
LTP treatment inhibited pro-inflammatory responses in HaCaT. LTP treatment into (**a**) TNF-α/IFN-γ-stimulated or LPS-stimulated HaCaT cells inhibited (**b**) IL-1β, (**c**) IL-6, (**d**) IL-8 and (**f**) IFN-γ gene expression. (**e**) IL-10 and (**h**) VEGFA gene expression increased by LTP treatment and (**g**) TGF-β1 gene expression was not regulated by LTP treatment in LPS- stimulated HaCaT cells. (**i**) Left panel: Western blot analysis showed that LTP treatment in HaCaT cells inhibited STAT3 activation, which is induced by IL-6 and involved in the pathogenesis of psoriasis. Cell lysates were separated on a 10% SDS-PAGE gel and transferred to an PVDF membrane. The membrane was cropped for the detection of target protein, followed by primary and secondary antibody incubation and visualization. Right panel: The graph showed the pSTAT3 intensities from three different independent experiments. Three independent experiments were performed. *P < 0.05, **P < 0.05.

To confirm the inhibitory effect of plasma on psoriasis-like skin inflammation, we determined the anti-inflammatory effect of LTP in keratinocyte cell lines because keratinocyte is the main cell type stimulated by imiquimod in this mouse model. HaCaT cells were activated with TNF-α and IFN-γ, which are involved in the pathogenesis of psoriasis, and the gene expression of IL-6, which participated in the Th17 cell differentiation and psoriasis, was measured by real-time PCR. Higher pro-inflammatory gene expression was induced in the activated HaCaT cells compared to unstimulated negative control HaCaT cells and LTP treatment inhibited the gene expression (Fig. 5a and Supplementary Figure 5). The inhibitory effect of LTP on IL-1β, IL-6, IL-8 and IFN-γ gene expression was also observed in the LPS-stimulated HaCaT cells (Fig. 5b–d and f). On the other hand, IL-10 and VEGFA gene expression increased by LTP treatment in LPS-stimulated HaCaT cells (Fig. 5e and h) while TGF-β1 gene expression was not modulated by LTP treatment (Fig. 5g). Furthermore, we determined whether LTP has an effect on STAT3 activation in HaCaT because STAT3 activation is crucial for Th17 cell differentiation and psoriasis pathogenesis. Figure 5i showed that LTP treatment inhibited STAT3 activation (pSTAT3) in IL-6-stimulated HaCaT cells. These results suggest that plasma inhibits STAT3 signaling pathway in keratinocytes, which could result in the inhibition of psoriasis in mice.

### LTP treatment enhances PD-L1 expression in keratinocyte

**Figure 6:**
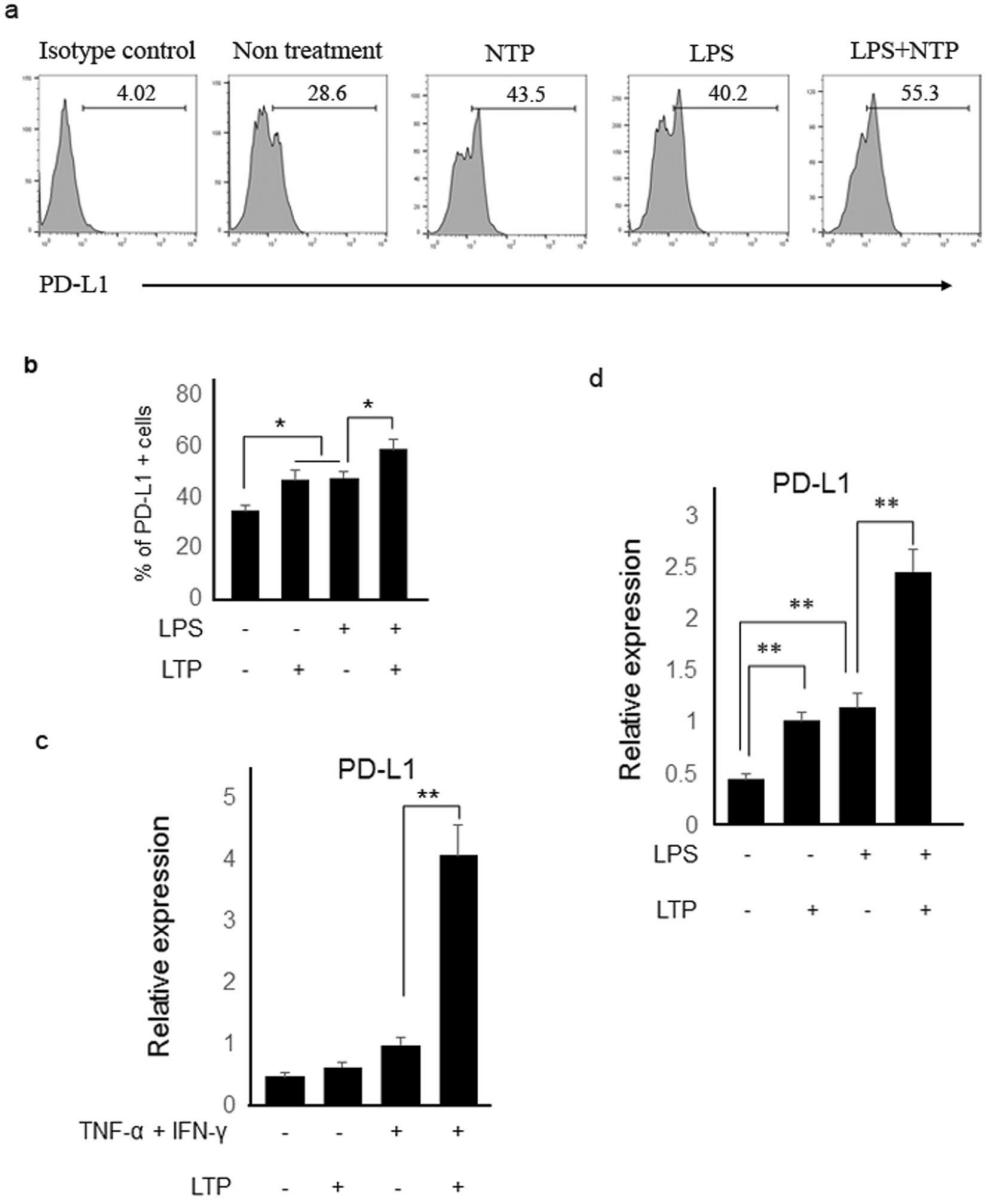
Plasma treatment enhanced PD-L1 expression in HaCaT cells. (**a**) LPS-stimulated and LTP-stimulated HaCaT cells induced higher PD-L1 expression than did the untreated HaCaT cells. LPS/LTP-stimulated HaCaT cells had more increased expression of PD-L1 than LTP only- or LPS only-stimulated cells. (**b**) The percentage of PD-L1 positive cells are indicated as a graph. Transcript levels of PD-L1 were also determined by real-time PCR. LTP treatment into (**c**) TNF- α/IFN-γ-stimulated or (**d**) LPS-stimulated HaCaT cells enhanced PD-L1 gene expression. Three independent experiments were performed. *P < 0.05, **P < 0.005.

It has been reported that PD-L1 expression in DC suppresses T cell activations through PD-1/PD-L1 binding. Thus, it has been proposed that over-expression or high expression of PD-L1 could induce inactivation of T cells and help to treat autoimmune diseases, in which unwanted CD4+ T cells are abnormally activated. Recently, it was reported that PD-L1 was highly expressed in the psoriasis patient skin. Furthermore, administration of recombinant PD-L1 ameliorated imiquimod-induced skin inflammation in mice. Therefore, we determined that plasma treatment could induce PD-L1 expression in HaCaT cells. As shown in Fig. 6a and b, LTP treatment induced PD-L1 expression in HaCaT cells and combination treatment with NTP and LPS induced more PD-L1 expression compared to a single treatment. RNA level of PD-L1 was also increased in TNFα/IFN-γ-stimulated and LPS-stimulated-HaCaT cells, and LTP treatment in the stimulated cells further enhanced the PD-L1 expression (Fig. 6c and d). These results suggest that plasma treatment may ameliorate psoriasis-like skin inflammation at least partially through regulation of PD-L1 gene expressions.

## Discussion

Plasma medicine is a novel area of study that combines plasma physics, biomedical science, and clinical medicine for the therapeutic application of physical plasma. The first clinical trial in patients for NTP was investigated in Germany in 2010 to reduce bacteria load on chronic wounds because NTP has an ability to kill bacteria, and chronic wounds are easily infected with bacterial pathogens, which results in the inhibition of the wound healing process^31^. Recently, a pilot study showed that NTP treatment could ameliorate psoriasis in patient although the mechanisms are unknown and the effect was not superior to that of conventional therapies^32^. Our study demonstrated that NTP treatment could inhibit psoriasis-like skin inflammation through suppression of immune responses using mouse model of psoriasis.

NTP is generated by the excitation of a gas in a discharge reactor and NTP contains various molecules including electrons, charged ions, reactive oxygen species (ROS), reactive nitrogen species (RNS), and UV. Thus, the effect of NTP could come from one of the molecules or mixture of the molecules. Although it is unknown which molecules are important for the effect of NTP, some reports have demonstrated that ROS has a protective role in immune-mediated diseases. For example, it was demonstrated that ROS could prevent imiquimod-induced psoriatic dermatitis^33^. It was also reported that hyperbaric oxygen therapy, which increases tissue levels of ROS, was effective in the treatment of chronic wound^34^, collagen-induce arthritis^35^, and Crohn’s disease^36^. On the other hand, some papers suggested that increased level of ROS might be involved in the pathogenesis of psoriasis^37,38^. Our results showed that NTP treatment inhibited expression of IMQ-induced NADPH oxidase 3 (NOX3), which catalyzes the production of superoxide, but NTP treatment had no effect on normal skin (Supplementary Fig. 7). Therefore, it seems that NTP treatment in psoriasis-like skin tissue in mice could ameliorate the disease partly through inhibition of ROS generation.

Psoriasis is a chronic inflammatory disease that affects 2% of the world’s population^39^ and affects patients socially and psychologically^40^. Thus, many experimental studies have been performed on the pathogenesis and treatment of psoriasis even though it is not a life-threatening disease. Currently, several treatments are available to treat psoriasis, such as phototherapy and immune suppressors (ciclosporin and methotrexate). However, phototherapy requires a heavy time commitment and might increase the risk of skin cancer. The side effects of immune suppressors are organ toxicities and increased risk of infections and cancer due to a suppressed immune system. Thus, a more efficient and safer treatment is necessary. Recently, ustekinumab, a targeted anti-IL-12/23 p40 monoclonal antibody, is used to treat psoriasis because the antibody inhibits the IL-23 signal pathway, which is critical to the pathogenesis of psoriasis. This antibody therapy is safer and effective compared to traditional treatment. It was also reported that increased PD-L1 expression could inhibit T cell activation. Thus, it might be possible that overexpression of PD-L1 could ameliorate psoriasis. Administration of recombinant PD-L1 inhibited psoriasis in mice^15^, implying that recombinant PD-L1 or anti-PD-1 antibody therapy might be useful in treatment of psoriasis. Biologic medications are relatively new type of treatment, which is used for many diseases, including rheumatoid arthritis, psoriasis, and cancer. However, biologic treatments are more expensive than conventional therapies, thus it is necessary to develop less expensive treatments. Our results showed that NTP treatment alleviates psoriatic phenotypes including skin redness, increased epithelial cell thickness, and enhanced pro-inflammatory cytokine and chemokine expressions (Figs 1 and 2), most likely through inhibition of Th17 cell differentiation (Fig. 3) and enhanced PD-L1 expression in keratinocytes (Fig. 6) even though LTP did not induce PD-1 expression in CD4+ T cells (data not shown). Because plasma treatment increases PD-L1 expression, it might be a substitute for the administration of recombinant PD-L1 or anti-PD-1 antibody therapy for the treatment of immune-mediated diseases. In addition, it would be interesting to investigate the effect of combination therapy with ustekinumab and plasma because mechanisms of ustekinumab’s action, which inhibits Th1 and Th17 cell differentiation, and of plasma, which induces PD-L1 expression and inhibits STAT3 activation (Fig. 5) are different. Thus, we expect that combination therapy might be more effective to inhibit Th17 cell activation and/or differentiation and result in a more effective treatment for psoriasis. The pathogenesis of other autoimmune diseases, such as multiple sclerosis, rheumatoid arthritis, and uveitis^19,41,42^ is also involved in IL-17/IL23 signal pathways. Some articles report that plasma could induce pro-inflammatory cytokines^43,44^. The researchers treated plasma 2 or 3 minute. Our experiment showed that NTP treatment for 1 minute and LTP (1 min plasma-treated medium) treatment into HaCaT cells did not induce pro-inflammatory cytokine expression (Supplementary Figure 6). This result indicates that increased cytokine and growth factor expression by CAP treatment (2 or 3 mimutes) might result from longer treatment time. Thus, duration of plasma treatment might be critical for the anti-inflammatory effect of plasma. Furthermore, we demonstrated that LTP also has an anti-inflammatory effect, suggesting that plasma could be used either directly or indirectly (in a plasma containing solution) for the treatment of diseases. Skin is the most easily accessible tissue for NTP application. Thus, NTP could be widely used for many other inflammatory skin disease treatments.

Currently, we are investigating how plasma treatment affects cellular responses. Preliminary result showed that plasma treatment into unstimulated normal HaCaT cells did not affect intracellular ROS level, but the plasma treatment increased ROS level in stimulated HaCaT cells, implying stimulated cells are more sensitive to plasma treatment. However, more studies are necessary to understand the cellular effect of plasma treatment.

In this study, we demonstrated for the first time that non-thermal atmospheric plasma can ameliorate psoriasis using a mouse model of psoriasis induced by imiquimod. The anti-psoriatic effect of plasma might result from the inhibition of inflammatory cytokine expression, CD4 T cell differentiation and induction of PD-L1 expression. These results might open up new application of plasma technology for the treatment of psoriasis and other autoimmune diseases.

## Materials and Methods

### Physical properties of N_2_-NTP

N_2_ gas was used as the source of non-thermal atmospheric plasma generation. Supplementary Figure 2 shows a schematic diagram describing the NTP producing machine. The properties of N_2_-based non-thermal atmospheric plasma was described previously^29^. Briefly, we performed optical emission spectrum analysis over a wide range of wavelengths from 280 nm to 920 nm with an optical emission spectroscope (SV2011, K-MAC, Korea) to identify the particles and radicals generated by the N_2_ plasma system. The emission spectrum of N_2_ plasma was mainly dominated by the presence of nitrogen species, containing N_2_ second (290–410 nm), first positive systems (600–700 nm) and N_2_^+^ first negative systems (410–600 nm). In addition, reactive radicals related with oxygen such as oxygen ion (O_2_^+^) at 500–600 nm and weak atomic nitrogen at 747, 822, 868 nm were detected. NO and NO_2_ do not emit light, so optical emission spectrum analysis cannot detect them. Thus, we confirmed the presence of NO_2_ in the plasma-treated PBS using a nitric oxide assay kit (Invitrogen) per the manufacturer’s protocols. The results showed that 60 µM of NO_2_ was made by a 1- minute treatment of the plasma into PBS. Maximum temperature was 37 °C at 1 cm distance. The temperature was below 30 °C at 3 cm distance.

### Generation of LTP

LTP was generated by treatment of non-thermal N_2_ plasma into culture media (RPMI 1640 or DMEM) for 30 (LTP 30) or 60 seconds (LTP 60 or LTP) per ml distant from the media (2 cm) (Supplementary Figure 2b). Cells were treated with LTP in all *in vitro* experiments. The cells were incubated with LTP for 6 h for detection of transcripts or 24 h for protein detection. HaCaT cells were incubated with LTP for 15 min for pSTAT3 detection.

### Mice

C57/BL6 mice were housed in an environmentally controlled room with a 12:12-hour light-dark cycle and free access to laboratory chow and water. Mice between 8 and 12 weeks of age were used. The protocol for mouse use was approved by the Committee for Ethics in Animal Experiments of Ajou University School of Medicine and performed in accordance with the institution guidelines.

### Cell line and reagents

The immortalized human keratinocyte cell line HaCaT was grown as monolayer cultures in DMEM (Welgene, Daegu, Korea) supplemented with 10% fetal bovine serum (Gibco, Carlsbad CA), 1% penicillin, and streptomycin (Gibco). Antibodies for STAT3, p-STAT3 and GAPDH were purchased from Cell Signaling Technology (Danvers, MA). All recombinant cytokines were purchased from PeproTech (Rocky Hill, NJ) and antibodies for CD4+ T cell differentiation were purchased from eBioscience (San Diego, CA). All antibodies for flow cytometry were purchased from Biogems (Westlake Village, CA) except anti-IL-17 antibody (eBIoscience).

### Imiquimod-induced psoriasis-like skin inflammation in mice

Imiquimod-induced psoriasis-like skin inflammation was induced as described previously^45^. Briefly, 62.5 mg of imiquimod cream (5%; 3.125 mg of the active compound) was applied on the shaved back skin of C57BL/6 mice every day for 5 days with or without NTP treatment for 1 minute on day 3 and 4 (*n* = 5 per group). The mice were sacrificed on day 5 for analysis.

### Immuno-histochemical analysis

Immuno-histochemistry was performed on paraffin-embedded skin tissue sections. The paraffin-embedded samples were sectioned at a thickness of 4μm and the sections were stained with anti-NOX3 antibody (Sigma-Aldrich, St. Louis, MO) overnight at 4 °C. The sections were rinsed in PBS and incubated for 2 h at room temperature with secondary antibody in SPlink HRP Detection Kit (GBI labs, Mukilteo, WA). After washing with PBS three times, the sections were stained with Liquid DAB+ Substrate Kit (GBI labs).

### Western blot analysis

Western blot analysis was performed as described previously^19^. Briefly, the cells and tissues were collected and lysed with RIPA buffer containing phosphate and a protease inhibitor cocktail on ice for 30 min. Following centrifugation at 14,000 g for 20 min at 4 °C. The proteins in supernatants were electrophoresed in 10% polyacrylamide gels and transferred to polyvinylidene fluoride membrane (Pall Corporation, NY). The membranes were blocked with 5% skim milk for 1–2 h and then incubated with primary antibody against STAT3, pSTAT3 and GAPDH (Cell Signaling) overnight. Next day, the membranes were washed and incubated with appropriate HRP-conjugated secondary antibodies. The signals were detected by an ECL kit (Amersham, Piscataway, NJ) and visualized by the Las detection program (Fujifilm, Japan).

### Flow cytometry

For the single cell suspensions, the back skins were collected and single cell suspensions were prepared as previously described^46^. Briefly, back skins were incubated for 45 min at 37 °C in RPMI 1640 containing Liberase. After incubation, the skins were disrupted mechanically. Cells were filtered through 40 μm nylon mesh and then collected, and stained with anti-CD3, anti-CD4, anti-CD11b, and anti-CD11c antibodies conjugated with fluorescence (BD PharMingen). Spleens and draining lymph nodes were collected and minced through 70 μm mesh for single cell suspensions. The cells were collected, washed and stimulated with plate-bound anti-CD3 (BD Pharmingen) and soluble anti-CD28 (eBioscience) antibodies in the presence of GolgiStop (BD Biosciences) for 5 h. Cells were harvested and intracellular staining was performed according to the manufacturer’s instructions (BD Pharmingen). The cells were acquired on a flow cytometer (FACS Calibur, BD Bioscience) and analyzed using FlowJo software (Ashland, OR).

### Generation of bone marrow-derived DC (BMDC) cells

CD11c^+^ cells were generated from bone marrow as previously described with minor modifications^47^. Briefly, bone marrow cells were obtained from tibias and femurs of 8- to 10-week-old C57BL/6 wild type mice, and lineage negative cells from the bone marrow cells were isolated using a lineage negative cell isolation kit according to the manufacturer’s protocol (Miltenyi Biotec). The lineage negative cells were grown with Dulbecco’s Modified Eagle’s Medium supplemented with 10% fetal bovine serum, granulocyte-macrophage colony-stimulating factor (10 ng/ml), and IL-4 (10 ng/ml) for 7 days. For activation of DCs, TNF-α (20  ng/ml) or lipopolysaccharide (1 μg/ml) was treated for 16 h.

### Isolation of primary CD4^+^ T cells

CD4^+^ T cells were purified from the lymph nodes and spleens of C57BL/6 mice. For the isolation of CD4^+^ T cells, lymph node cells and splenocytes were loaded onto T-cell enrichment columns (R&D systems), and the elutant was used to purify CD4^+^ T cells using a CD4^+^ CD62L^+^ T cell isolation kit as specified by the manufacturer (Miltenyi Biotec, Bergisch-Gladbach, Germany).

### CD4+ T cell differentiation

Purified naïve CD4+ T cells were cultured under Th1 or Th17 cell differentiation condition. For Th1 cell differentiation, naïve CD4+ T cells were cultured with plate-bound anti-CD3, soluble anti-CD28 (5 µg/ml) and anti-IL-4 (10 μg/m.) antibodies (eBioscience) combined with recombinant IL-12 (10 ng/ml) (Peprotech) in 96 well plates (BD Pharmingen). For Th17 cell differentiation, naïve CD4+ T cells were cultured with plate-bound anti-CD3 (10 μg/ml), soluble anti-CD28 (5 μg/ml), anti-IFN-ϒ (10 μg/ml) and anti-IL-4 (10 μg/ml) antibodies (eBioscience) combined with recombinant TGF-β1(5 ng/ml) and IL-6 (10 ng/ml) (PeproTech) in 96 well plates (BD Pharmingen). The CD4+ T cells were cultured with or with LTP for 4 days and collected for RNA isolation.

### RNA isolation, cDNA synthesis, and quantitative real-time PCR

Total RNA isolation and the first strand synthesis of cDNA was performed described previously with minor modifications^37^. Briefly, Total RNA was isolated from CD4+ T cells or mouse skin with the TRIzol reagent (Invitrogen, Carlsbad, CA). The first strand of cDNA was synthesized from 1 μg of total RNA using a reverse transcription system (Toyobo, Japan). The primer sets for IL-6, IL-17, TNF-α, CXCL1, CCL20, PD-L1 and GAPDH were purchased (Qiagen, Hilden, Germany). GAPDH mRNA was used as an endogenous control. PCR was performed using Step One Plus Real-Time PCR System (Applied Biosystems, Foster City, CA) and the SYBR Green PCR Kit (Applied Biosystems). The amplification program consisted of 1 cycle of 95 °C for 10 min, followed by 40 cycles of 95 °C for 20 sec, 55 °C for 20 sec, and 72 °C for 20 sec.

### Statistical analysis

Data are presented as mean ± standard error of mean (s.e.m.) of three independent experiments and statistical comparisons between groups were performed using unpaired 2-tailed *t*-tests. *P*-values < 0.05 were considered statistically significant.

## Electronic supplementary material

Supplementary figures and table
