# Supplementary material for: Non-thermal atmospheric plasma ameliorates imiquimod-induced psoriasis-like skin inflammation in mice through inhibition of immune responses and up-regulation of PD-L1 expression
Source: Sci Rep. 2017 Nov 14;7:15564. doi: 10.1038/s41598-017-15725-7 (PMC5686068; doi:10.1038/s41598-017-15725-7)
Supplement: Supplementary file 1 — Supplementary figures and table [file 41598_2017_15725_MOESM1_ESM.pdf]

Non-thermal atmospheric plasma ameliorates imiquimod-induced psoriasis-like skin inflammation in mice through inhibition of immune responses and up-regulation of PD-L1 expression.

Yun Sang Lee[1], Myung-Hoon Lee[1,2], Hang-Jun Kim[1,2], Ho-Ryun Won[1], Chul-Ho Kim[1,2]

[1]Department of Otolaryngology, School of Medicine, Ajou University, Suwon, Republic of Korea, [2]Department of Molecular Science and Technology, Ajou University, Suwon, Republic of Korea

Supplementary Figure 1

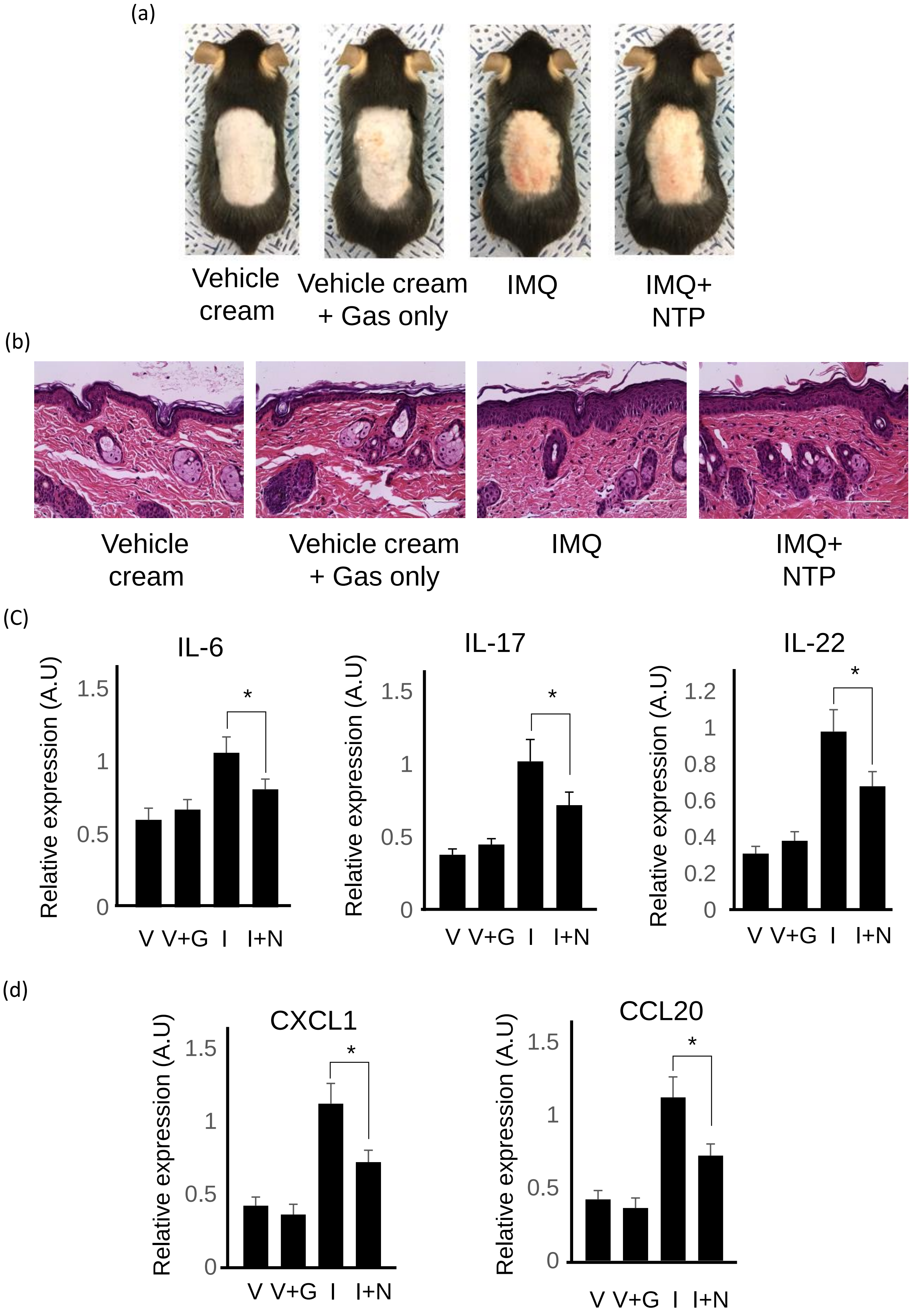

Supplementary figure 1. (a) Vehicle cream (Vaseline Lanette cream) and vehicle cream + gas only-treatment did not affect skin inflammation. (b) H&E staining (c) cytokine (d) chemokine gene expression was not effected by vehicle cream and N2 gas.

Supplementary Figure 2

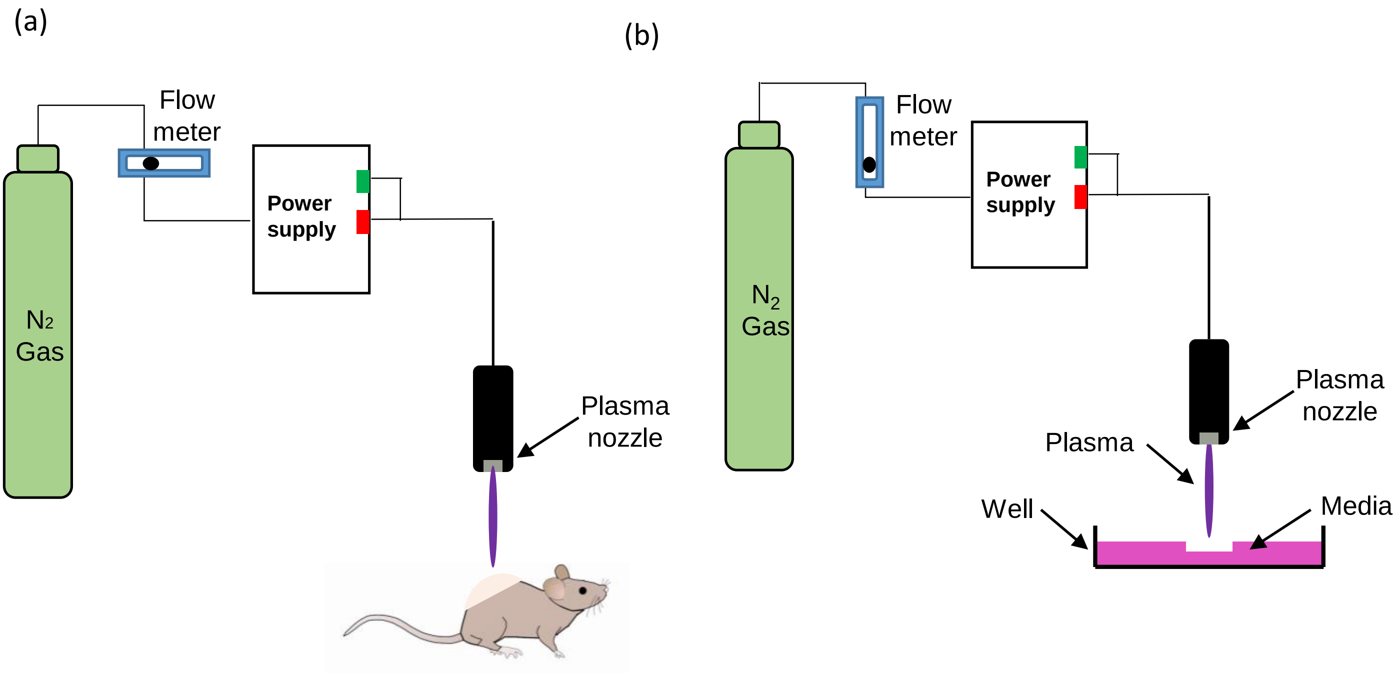

Supplementary figure 2. A schematic diagram describing treatment of mouse with the NTP producing machine (left figure) and the method of making LTP (right figure). Non-thermal plasma was treated for 30 or 60 seconds per ml and used for in the vitro experiments.

Supplementary Figure 3

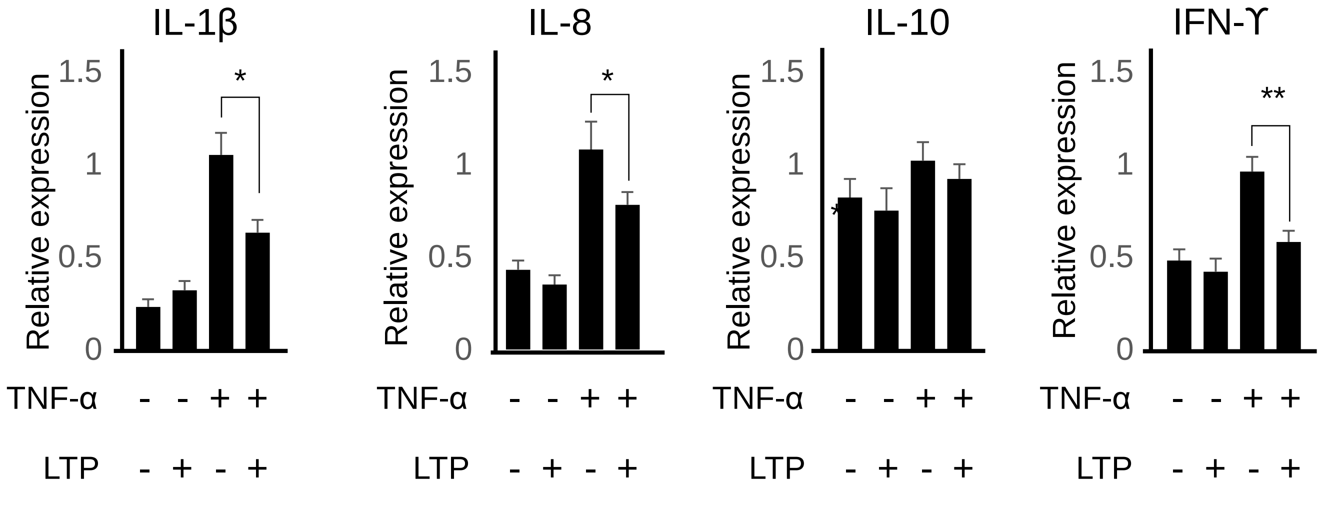

Supplementary Figure 3. LTP treatment inhibited TNF- α-induced proinflammatory cytokine expression in BMDC

Supplementary Figure 4

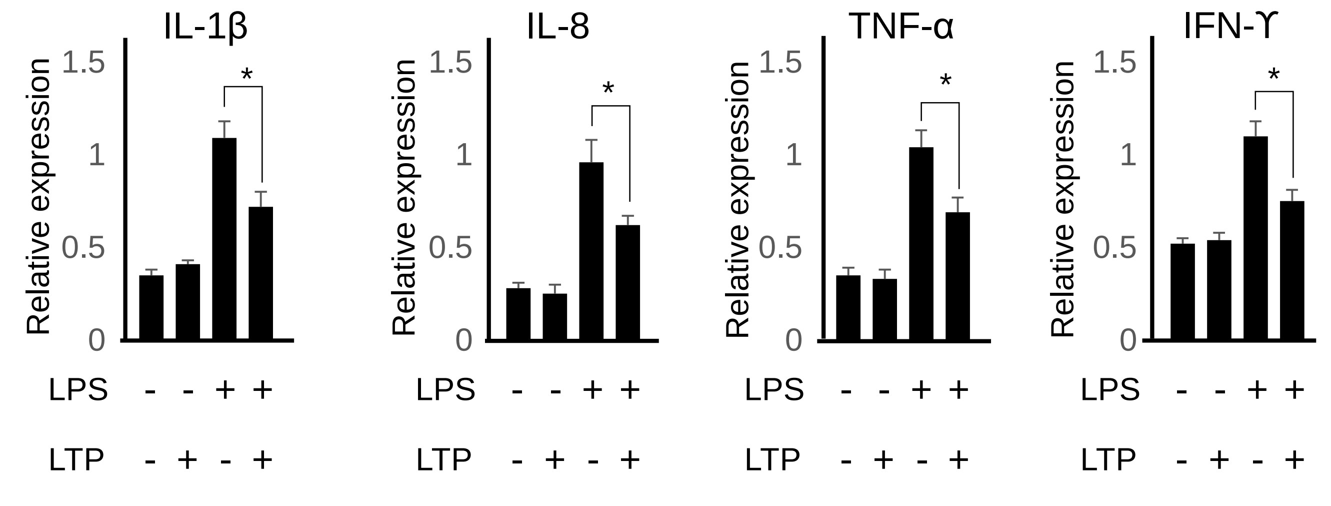

Supplementary Figure 4. LTP treatment inhibited LPS-induced proinflammatory cytokine expression in BMDC

Supplementary Figure 5

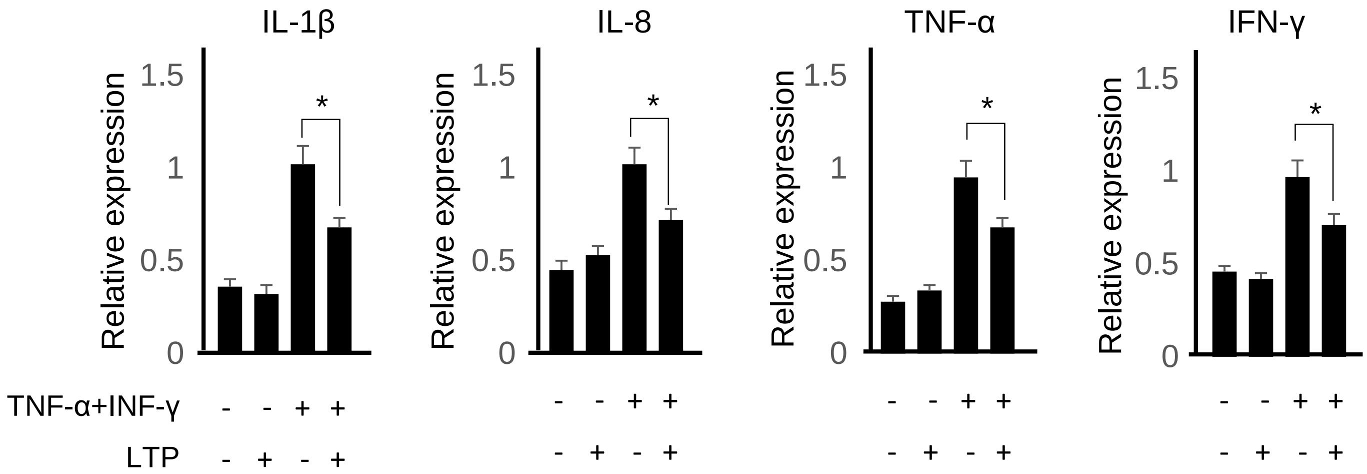

Supplementary Figure 5. LTP treatment inhibited TNF- α/IFN- γ induced proinflammatory cytokine expression in HaCaT cells

Supplementary Figure 6

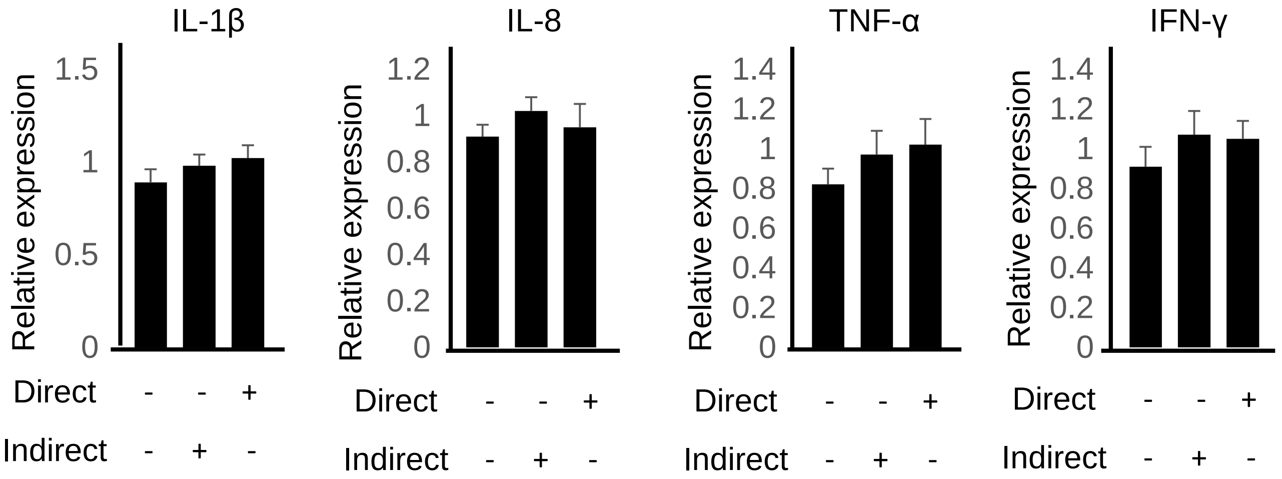

Supplementary Figure 6. Direct and indirect plasma treatment did not affect gene expression in unstimulated normal HaCaT cells

Supplementary Figure 7

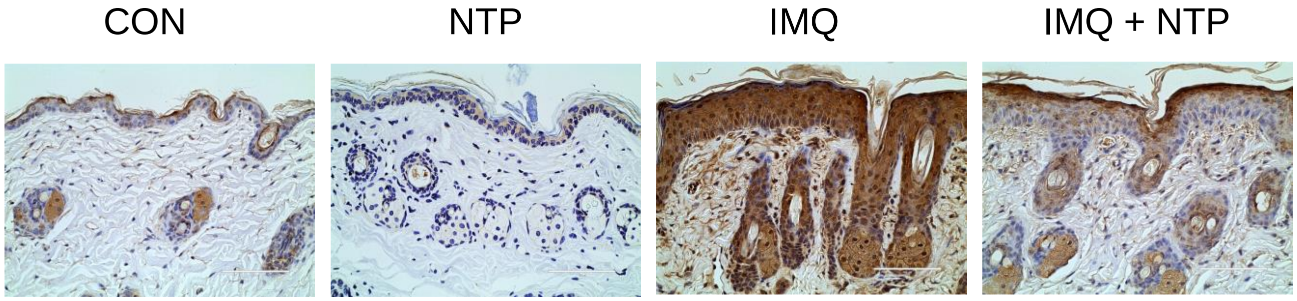

Supplementary Figure 7. Treatment with NTP inhibited IMQ-induced NOX3 expression in mouse

Supplementary table 1

Primer sequences for real time PCR

hIL-1β: forward 5'ACAGATGAAGTGCTCCTTCCA3'
reverse 5'GTCGGAGATTCGTAGCTGGAT3'

hIL-8: forward 5'ATGACTTCCAAGCTGGCCGTGGCT3'
reverse 5'TCTCAGCCCTCTTCAAAAACTTCT3'

hIL-10: forward 5' CATCGATTTCTTCCCTGTGAA3'
reverse 5' TCTTGGAGCTTATTAAAGGCATTC3'

hIFN-ϒ: forward 5'CTAATTATTCGGTAACTGACTTGA3'
reverse 5'ACAGTTCAGCCATCACTTGGA3'

hTGF-β1: forward 5'CCCAGCATCTGCAAAGCTC3'
reverse 5'GTCAATGTACAGCTGCCGCA3'
